# Impact of type 1 diabetes on the composition and functional potential of gut microbiome in children and adolescents: possible mechanisms, current knowledge, and challenges

**DOI:** 10.1080/19490976.2021.1926841

**Published:** 2021-06-08

**Authors:** Pari Mokhtari, Julie Metos, Pon Velayutham Anandh Babu

**Affiliations:** Department of Nutrition and Integrative Physiology, College of Health, University of Utah, Salt Lake City, Utah, USA

**Keywords:** Composition and functional potential, gut microbiome, type 1 diabetes, children, adolescents, gut dysbiosis

## Abstract

Diabetes prevalence and incidence among youth have been increasing globally. Type 1 Diabetes (T1D) in children or adolescents accounts for 5–10% of all diagnosed cases of diabetes. Emerging evidence indicates that genetic factors, especially genes in the human leukocyte antigen region, are not the only factors involved in the predisposition of an individual to T1D. The pathogenesis and development of T1D is driven by both genetic predisposition and environmental factors. Studies indicate that gut microbiota is one of the potential environmental influencers involved in the pathophysiology of TID. Gut microbiota mediates the development of diabetes by altering intestinal permeability, modifying intestinal immunity, and molecular mimicry. The gut microbial diversity, taxonomic profile, and functional potential of gut microbes are significantly altered in individuals with T1D as compared to healthy individuals. However, studies are still needed to identify the specific microbes and microbial metabolites that are involved in the development and pathogenesis of T1D. This will help the development of microbiome-based therapeutic strategies for the prevention and treatment of T1D. The present review article highlights the following: (i) the current knowledge and knowledge gaps in understanding the association between T1D and gut microbiome specifically focusing on the composition and functional potential of gut microbiome in children and adolescents, (ii) the possible mechanisms involved in gut microbiome-mediated T1D pathogenesis, and (iii) challenges and future direction in this field.

**Abbreviations:** B/F ratio: Bacteroidetes to Firmicutes ratio; F/B ratio: Firmicutes to Bacteroidetes ratio; FDR: First-degree relatives; GPR: G protein-coupled receptors; HLA: human leucocyte antigen; IL: interleukin; IFN- γ: interferon-γ; KEGG: Kyoto Encyclopedia of Genes and Genomes; LPS: lipopolysaccharide; mTOR: mammalian target of rapamycin; PICRUSt: Phylogenetic Investigation of Communities by Reconstruction of Unobserved States; SCFA: short chain fatty acids; T1D: Type 1 diabetes; T2D: Type 2 diabetes; TJ: tight junction; T_regs_: regulatory T cells.

## INTRODUCTION

Diabetes is a chronic progressive autoimmune disease that imposes a substantial clinical and health burden due to the challenges of disease management and the risks of numerous associated complications.^1,2^ Diabetes prevalence and incidence among youth have been increasing globally.^1,2^ SEARCH is a multicenter study for diabetes in youth in the United States that has aimed to learn more about diabetes among children and adolescents since 2000. The results of this study in 2011–2012 indicated an annual relative increase of 1.8% and 4.8% from 2002 to 2011–2012 for Type 1 diabetes mellitus (T1D) and Type 2 diabetes mellitus (T2D), respectively.^2^ Approximately, 193,000 of the 23 million people diagnosed with diabetes in 2015 were children and adolescents younger than age 20.^2^ The International Diabetes Federation revealed that more than one million subjects younger than 20 years old were affected by T1D in 2017 and approximately 86,000 children were diagnosed with T1D every year.^3^ Indeed, T1D onset occurs in children or adolescents accounts for 5–10% of all diagnosed cases of diabetes.^4^

**Table 1.** Impact of type 1 diabetes on the composition of gut microbiota in children and adolescents

Country and participants	Diversity	Abundance at phyla level	Abundance at class/order/family level	Abundance at genus level	Abundance at species level
Finland: Children who developed autoimmunity and T1D over time, and age matched healthy control with similar genotype who did not become autoimmune during the study (n = 3).^58^	↓ T1D	↑ Bacteroidetes ↓ Firmicutes	Class ↑ Bacteroidia ↓ Clostridia Order ↑ Bacteroidales ↓ Clostridiales Family ↑ Veillonellaceae ↑ Bacteroidaceae ↑ Rikenellaceae	↑ *Bacteroides* ↓ *Eubacterium* ↓ *Faecalibacterim*	↑ *Bacteroides ovatus* ↓ *Bacteroides vulgatus* ↓ *Bacteroides ragilis* ↓ *Firmicute CO19*
Finland: Children tested positive for at least two diabetes-associated autoantibodies and control children matched for age, sex, and HLA-DQB1 genotype (n = 18).^57^	↓ Auto-antibody positive children	↑ Bacteroidetes	Family ↑ Bacteroidaceae	↑ *Bacteroides*	↓ *Bifidobacterium adolescentis* ↓ *Faecalibacterium prausnitzii* ↓ *Clostridium clostridioforme* ↓ *Roseburia faecis* ↑ *Clostridium perfringens* ↓ *Eubacterium hallii*
Spain: Children with T1D and healthy children (n = 16).^45^		↓ Actinobacteria ↓ Firmicutes ↑ Bacteroidetes	Group ↓ Blautia coccoides-Eubacterium rectale	↑ *Veillonella* ↑ *Clostridium* ↑ *Bacteroides* ↓ *Prevotella* ↓ *Bifidobacterium* ↓ *Lactobacillus*	
Turkey: Children with T1D and healthy children (n = 35).^75^			Family ↑ Enterobacteriaceae	↓ *Lactobacillus* ↓ *Bacteroides* ↓ *Biﬁdobacterium*	↓ *Biﬁdobacterium spp*
Germany: Children with anti-islet cell autoantibodies positive and islet cell autoantibody – negative (n = 22).^63^				↓ *Veillonella* ↑ *Enterococcus* ↑ *Sarcina* ↑ *Prevotella* ↑ *Corynebacterium* ↑ *Barnesiella* ↑ *Candidatus Nardonella* ↓ *Staphylococcus* ↓ *Nocardioides*	
dxxm 28 diabetic childrenxm 28 diabetic children Finland, France, Greece, Estonia and Lithuania: Children with T1D (n = 28) and age matched control (n = 27).^39^	↑ Older diabetic children	↓ Clostridium cluster XIVa ↓ Clostridium cluster IV ↑ Bacteroidetes ↑ Streptococci ↑ Clostridium stercorarium		↑ *Streptococcus mitis et rel*	
USA: Children newly diagnosed with T1D (n = 35), individuals with 1–4 autoantibodies (n = 21), seronegative first-degree relatives (FDR) of subjects with islet autoimmunity (n = 32), and unrelated healthy controls (n = 23).^65^		↑ Firmicutes (seropositive subjects) ↑ Bacteroidetes (seropositive subjects)	Family ↑ Prevotellaceae (seropositive)	↑ *Catenibacterium* (seropositive) ↓ *Succiniclasticum* (new-onset subjects) ↓ *Catenibacterium* (new-onset subjects) ↑ *Bacteroides* (subjects with multiple autoantibody) ↑ *Akkermansia* (subjects with multiple autoantibody) ↓ *Prevotella* (subjects with multiple autoantibody) ↓ *Staphylococci* (new-onset, seropositive, and seronegative FDRs) ↓ *Lactobacilli* (new-onset, seropositive, and seronegative FDRs)	
Italy: Children with β-cell autoimmunity at risk for T1D and healthy children (n = 10).^52^					↑ *Gemella sanguinis* ↑ *Dialister invisus* ↑ *Bifidobacterium longum*
Finland and Estonia: Infants genetically predisposed to T1D (n = 33) and healthy controls (n = 22).^59^					↑ *Blautia* ↑ *Ruminococcus*
Azerbaijan, Jordan, Nigeria and Sudan: Children/adolescents newly diagnosed with T1D (n = 73), and age and location matched controls (n = 104).^64^		↑ Proteobacteria ↓ Firmicutes	Class ↑ Gamma-proteobacteria ↓ Clostridia	↑ *Escherichia* ↓ *Eubacterium* ↓ *Roseburia* ↓ *Clostridium cluster IV* ↓ *Clostridium cluster XIVa*	
China: Children with T1D and healthy children (n = 15).^27^	↓ T1D		Order ↓ Micrococcales Family ↓ Pasteurellaceae ↓ Caulobacterales	↑ *Blautia* ↓ *Haemophilus* ↓ *Lachnospira* ↓ *Dialister* ↓ *Acidaminococcus* ↓ *Intestinimonas*	
Spain: Children with T1D (n = 15), children with MODY2 (n = 15), and healthy children (n = 13).^47^		↑ Bacteroidetes ↓ Firmicutes ↓ Actinobacteria ↓ Proteobacteria	Family ↑ Bacteroidaceae ↑ Rikenellaceae ↑ Prevotellaceae ↑ Ruminococcaceae ↑ Veillonellaceae ↑ Streptococcaceae ↑ Enterobacteriaceae	↑ *Bacteroides* ↑ *Ruminococcus* ↑ *Veillonella* ↑ *Blautia* ↑ *Streptococcus* ↑ *Sutterella* ↑ *Enterobacte* ↑ *Prevotella* ↓ *Bifidobacterium* ↓ *Roseburia* ↓ *Faecalibacterium* ↓ *Anaerostipes* ↓ *Lachnospira*	
Portugal: Children with T1D and healthy children (n = 3).^48^					↑ Bacterial proteins from - *Eubacterium rectale* - *Faecalibacterium prausnitzii* - *Bacteroides dorei* - *Bacteroides uniformis*
Italy: Children with T1D and healthy children (n = 13).^61^	↓ T1D			↓ *Eubacterium*	↑ *Bacteroides clarus* ↑ *Alistipes obesi* ↑ *Bifidobacterium longum* ↑ *Methanobrevibacter smithii* ↓ *Bacteroides vulgatus* ↓ *Bacteroides oleiciplenus* ↓ *Bacteroides coprophilus* ↓ *Bacteroides doreias* ↓ *Faecalibacterium prausnitzii* ↓ *Fusicatenibacter saccharivorans*
Finland: Seroconverted to T1D-related autoimmunity (n = 29 and 22 children out of 29 later developed T1D) and healthy children (n = 47).^69^		↑ Bacteroidetes		↑ *Bacteroides*	↑ *Bacteroides dorei* ↑ *Bacteroides vulgatus*
Mexico: T1D at onset (n = 8), T1D after 2 years treatment (n = 13), and healthy controls (n = 8).^46^				↑ *Bacteroides* ↓ *Prevotella* ↓ *Megamonas* ↓ *Acidaminococcus*	
Finland: Children with two autoimmune antibodies and corresponding controls (n = 4).^44^		↑ Actinobacteria ↑ Bacteroidetes ↑ Proteobacteria		↑ *Bacteroides* ↑ *Lactobacillus* ↑ *Lactococcus* ↑ *Bifidobacterium* ↑ *Streptococcus* ↑ *Veillonella* ↑ *Alistipes* ↓ *Prevotella* ↓ *Akkermansia*	
Finland, Estonia and Russia: Infants selected on the basis on similar HLA risk class distribution which includes high, moderate, slightly increased, and neutral/protective (n = 74 infants from each country).^73^			Family ↑ Gemellaceae	↑ *Rothia* ↓ *Bilophila* ↓ *Sutterella*	
Finland: Infants and toddlers with early-onset islet autoimmunity followed by type 1 diabetes, and matched controls (n = 18).^67^			Class ↓ Bacteroidia		↓ *Bacteroides dorei* ↓ *Bacteroides vulgatus* ↓ *Bacteroides caccae* ↓ *Bifidobacterium bifidum* ↓ *Bifidobacterium pseudocatenulatum*
Sweden: ABIS cohort (All Babies in Southeast Sweden), a prospective population-based cohort study including all children born in southeast Sweden. One year stool samples of 403 individual were analyzed.^15^				*Intestinibacter* *Romboutsia* (Associated with lower genetic risk HLA genotypes)	
USA, Finland, Germany and Sweden: The Environmental Determinants of Diabetes (TEDDY) study collected samples monthly from three months of age until the clinical end point which include islet autoimmunity or T1D . Healthy control (n = 415), seroconverted but T1D not diagnosed (n = 267) and T1D diagnosed (n = 101).^23^		↑ Proteobacteria		↑ *Streptococcus*	↑ *Streptococcus mitis* ↑ *Streptococcus oralis* ↑ *Streptococcus mitis pneumoniae* ↑ *Bifidobacterium pseudocatenulatum* ↑ *Roseburia hominis* ↑ *Alistipes shahii*
Filand and Estonia: New borns with HLA Dr-DQ positive were followed-up until age of 3 years and stool samples collected monthly (n = 33). Two positive autoantibodies seroconversion during their followup and did not develop T1D (n = 11) and children progressed to T1D (n = 4) and healthy healthy controls mached for gender, HLA genotype and country (n = 22).^62^			Class: An inhibitory effect from Gammaproteobacteria to Bacteroidia in the T1D progressors.		

T1D is an autoimmune disorder characterized by an absolute insulin deficiency caused by the immune cell-mediated destruction of ß- cells in the pancreas.^5^ The loss of ß- cells which produce insulin results in a life-long exogenous insulin dependency and the development of T1D.^5^ Recent studies indicate that genetic factors especially genes in the human leukocyte antigen (HLA) region is not the only factor involved in the predisposition of an individual to T1D and acceleration of diabetes.^6,7^ Studies conducted on identical twins show that only a fraction of the individuals who were genetically predisposed will develop Type 1 diabetes.^8,9^ As genetic susceptibility alone is not enough to explain the casualty of T1D, the pathogenesis and development of the autoimmune disease is thought to be majorly driven by both genetic predisposition and environmental factors.^10–14^ The intestinal microbiome which is known to interact with and influence the immune system of the host has drawn a considerable interest as a potential environmental influencer of T1D.^15^

The human body contains trillions of bacterial, viral, and fungal microorganisms. The cumulative size of the microbial ‘meta-genome’ in the human gut is 500-fold greater than the human genome.^16,17^ The microbiota and mammalian host intimate coevolution has created a symbiotic relationship for hundred thousands of years that contributes to a multitude of crucial physiological functions of the healthy human body including metabolic signaling, regulation of gut barrier integrity and mobility, nutritional function, energy metabolism, immune system development, and brain function.^18,19^ The host lifestyle, diet, age, gender, geographical location, genetic background, hygiene, antibiotic use, and other medical practices results in extensive alterations in the diversity of the microorganisms.^6,20,21^ These factors play a pivotal role in modifying the composition and function of the microbiome that impacts the immune and metabolic systems, thereby contributing positively or negatively to the risk of T1D.^6,22,23^ Patients with diabetes have distinct gut microbiota in comparison to healthy individuals that are linked to changes in intestinal permeability, inflammation, and insulin resistance.^24^

Gut microbiome dysbiosis is recognized as one of the major contributors to the development of diabetes.^6,7,24^ “Dysbiosis” is characterized by the imbalance of the gut microbiota which means lower microbial diversity, loss of beneficial microorganism and/or the expansion of potentially harmful microorganisms.^25,26^ Studies show that gut dysbiosis contributes to immune dysfunction, metabolic disorders, insulin resistance, T1D, T2D, obesity, inflammatory bowel disease, and celiac disease.^27–29^ Despite the recent developments in the field of the gut microbiome, there are still critical knowledge gaps in understanding the gut microbiome in children and adolescents with T1D and need further investigations. There are few review articles published on gut microbiome and T1D but the present review article is different from them in a number of ways. The present review article will focus on (i) the current knowledge and knowledge gaps in understanding the association between T1D and gut microbiome specifically focusing on the composition and functional potential of gut microbiome in children and adolescents, (ii) the possible mechanisms involved, (iii) challenges, and (iv) future direction.

## REVIEW STRATEGY

**Figure 1.**
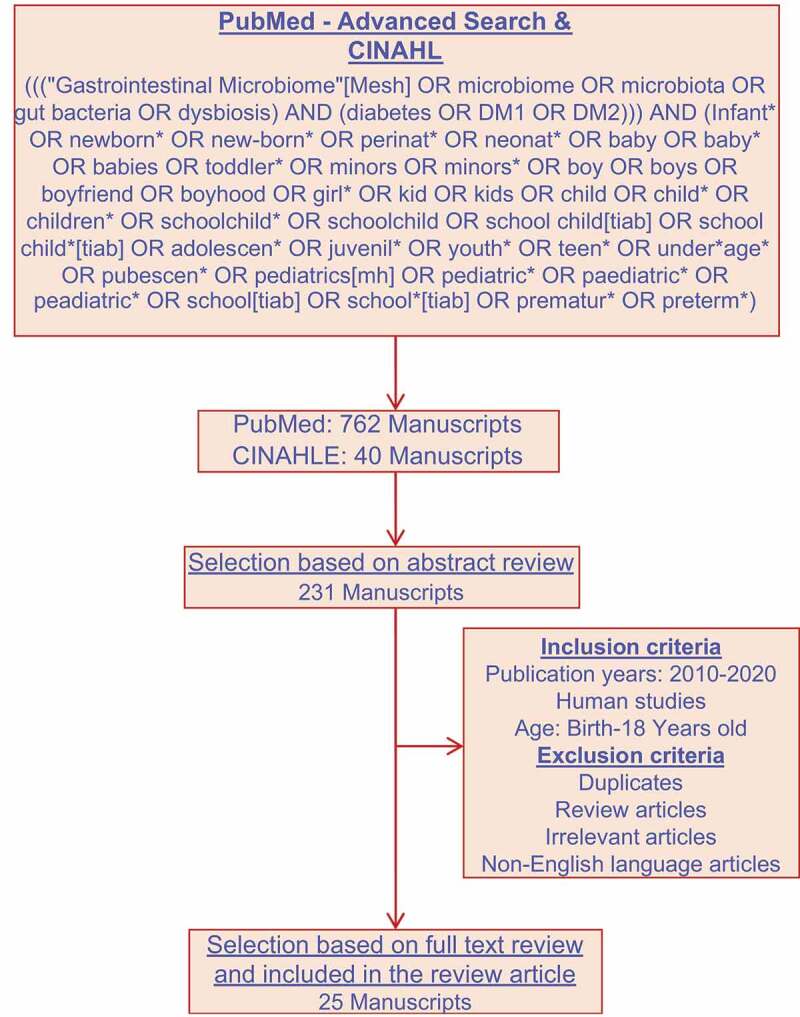
Review strategy

A literature search was conducted using electronic databases such as PubMed and CINAHL to identify the research manuscripts for this review article that is focused on identifying the impact of gut microbiome on T1D in children and adolescents. The following keywords were used in the PubMed advanced search builder: (((“Gastrointestinal Microbiome”[Mesh] OR microbiome OR microbiota OR gut bacteria OR dysbiosis) AND (diabetes OR DM1 OR DM2))) AND (Infant* OR newborn* OR new-born* OR perinat* OR neonat* OR baby OR baby* OR babies OR toddler* OR minors OR minors* OR boy OR boys OR boyhood OR girl* OR kid OR kids OR child OR child* OR children* OR schoolchild* OR schoolchild OR school child[tiab] OR school child*[tiab] OR adolescen* OR juvenil* OR youth* OR teen* OR under*age* OR pubescen* OR pediatrics[mh] OR pediatric* OR paediatric* OR peadiatric* OR school[tiab] OR school*[tiab] OR prematur* OR preterm*). We used a validated pediatric search filter with high sensitivity in this review. Pubmed database provided 762 manuscripts and CINAHLE data base provided 40 manuscripts. Based on the abstract review, 231 manuscripts were selected out of these 802 manuscripts. The inclusion criteria for this review article include (i) articles published over the past 10 years 2010–2020, (ii) human studies, and (iii) the age range of birth to 18 years old. The exclusion criteria include (i) duplicates, (ii) review articles, (iii) irrelevant articles, and (iv) the articles published in a language other than English. Based on the inclusion and exclusion criteria 25 research manuscripts were selected out of 231 manuscripts. These 25 research manuscripts that investigated the impact of T1D on the composition and functional potential of gut microbiome in children and adolescents were selected for this review article (Figure 1).

## DATA EXTRACTION

The following variables were extracted independently by two authors: article title, authors, publication year, country, study design, participants age, gender/race, the number of participants, type of diabetes (T1D/T2D), blood parameters (blood glucose/HbA1C), analysis technique, microbiome diversity, relative abundance of microbiome at different taxonomic levels, analysis of short chain fatty acids (SCFA), possible mechanisms, gut permeability assessment, and functional analysis (predictive or actual metagenomics).

## ROLE OF GUT MICROBIOME IN THE DEVELOPMENT OF TYPE 1 DIABETES AND THE POSSIBLE MECHANISMS INVOLVED

**Figure 2.**
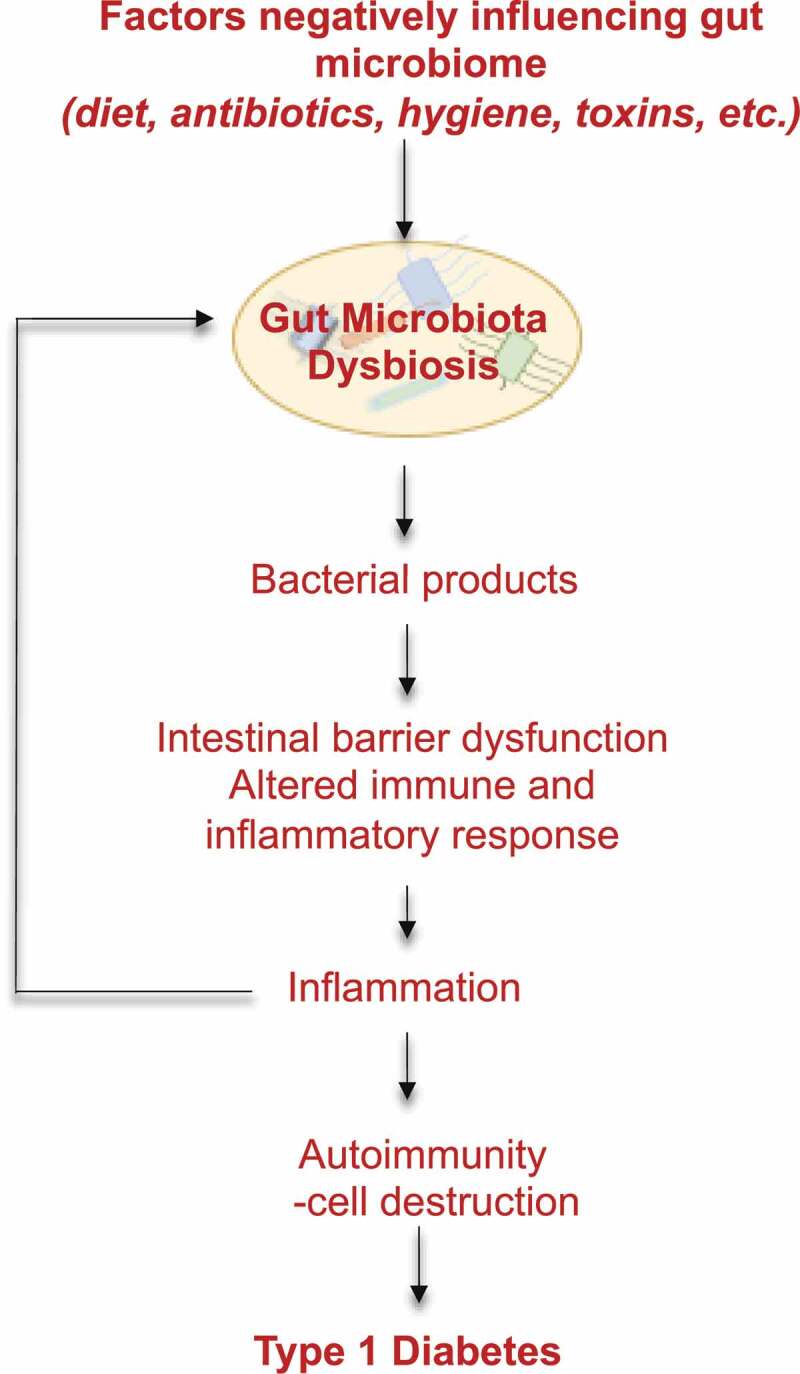
Gut microbiota dysbiosis influence type 1 diabetes

Emerging evidence from animal and human studies indicate that gut microbiota and its products are involved in the pathophysiology of TID.^9^ Gut microbiome appears to play a pivotal role in the development of diabetes by altering intestinal permeability, modifying intestinal immunity, and molecular mimicry in which microbial antigens can trigger autoimmunity by mimicking self-antigens due to sequence similarity between microbial peptides and autoantigens (Figure 2).^30^

### Intestinal barrier dysfunction

Gut microbiome dysbiosis is defined as the major shifts in microbial community composition associated with a loss of important physiological functions. Dysbiosis leads to adverse effects on human health, and it has been implicated in T1D pathogenesis.^31^ Human and animal studies indicate the role of ‘leaky gut’ in the progression and the development of T1D.^32–34^ An increased intestinal permeability as a consequence of an impaired intestinal integrity allows dietary antigens and immune stimulants such as exogenous antigens and the microbial components to translocate into the circulation.^35^ This can promote systemic inflammation and autoimmune progression leading to the destruction of the pancreatic β-cells.^32,36^ Gut microbiome can modulate gut integrity through the formation of SCFAs.^76^ SCFAs such as butyrate, acetate, and propionate are produced when non-digestible carbohydrates such as dietary fibers undergo bacterial fermentation.^76,37^ Emerging evidence indicate that butyrate-producing microbes are negatively correlated with the risk of T1D.^38,39^ Butyrate plays a pivotal role in maintaining intestinal integrity and intestinal epithelial cell growth. Butyrate reduces gut permeability, inhibits bacterial translocation, and possesses anti-inflammatory properties.^40^ It regulates the assembly of tight junction (TJ) by affecting the expression of TJ proteins which include claudin2, cingulin, occludin, and zonula occludens.^41,42^ TJ and its associated proteins contribute to the construction of intestinal tight junction barrier.^43^ Indeed, disrupted intestinal epithelial TJ barrier is reported in the development of a number of diseases.^43^ Conversely, propionate, acetate, and succinate producing bacteria such as Bacteroides are shown to be positively associated with the development of T1D.^39,44–48^ These bacteria can reduce the TJ assembly, which results in a consequential increase in gut permeability and eventually promoting T1D-associated autoimmunity.^44,49^

Although the mechanisms by which the gut microbiota modulates intestinal permeability and epithelial barrier function remain unclear, higher levels of Zonulin is one of the proposed mechanisms for the impaired gut integrity mediated metabolic disorders. Zoulin is a physiological modulator and a fundamental regulator of intercellular tight junctions and intestinal barrier function in metabolic disorders.^47,50^ Zoulin as an epithelial cell tight junction’s molecule sense microorganism toxins and microbial antigens that consequently increase bacterial translocation and gut permeability. The Zonulin production is up-regulated into the gut lumen due to imbalanced bacterial colonization.^51^ The receptors localized on the surface of the intestinal epithelial cells recognize the released zonulin and provoke alterations in the TJ dynamics.^32^ These changes in TJ dynamics compromise Zoulin-1 and occluding phosphorylation and cytoskeleton remodeling^32,51^ Thus the disassembly of TJ results in an increased gut permeability.^32^ Aberrant microbial composition, overabundance of opportunistic pathobionts, decreased microbial diversity, decreased butyrate-producer and mucin-degrader bacteria lead to disruption of the intestinal barrier integrity with an increase in gut permeability which can lead to the pathogenesis of T1D.

Only limited studies assessed the gut permeability in children with diabetes. A recent study evaluated the gut permeability by measuring the plasma level of Zonulin and showed an increased gut permeability in children with T1D.^47^ Another study assessed the gut permeability in T1D using lactulose/mannitol tests, which involved the administration of an oral dose of probes (lactose and mannitol) and measuring their urinary excretion after 5 hours.^52^ This study reported a significant increase in urinary lactulose in children at risk for T1D compared to healthy controls. These evidence indicate that the altered microbial composition and elevated gut permeability are simultaneously related to pre-pathological condition of T1D.

### Altered immune and inflammatory response

Gut microbiota is an essential part of the healthy mammalian immune system development.^53^ Indeed, the crucial role of the gut microbiota in maturation of the host immune system is becoming increasingly evident.^32,53^ The gut microbiota continuously educates the immune system to discriminate between commensal and pathogenic bacteria, and as a consequence, pathogenic bacteria provoke a pro-inflammatory response while commensal bacteria an anti-inflammatory response.^54^ The gut microbiota promotes the differentiation and expansion of key mediators of immune tolerance such as regulatory T cells (T_regs_).^54^ The gut microbiome affects T cell differentiation through modulating the neutrophils migration and function.^54^ The formation of SCFAs such as butyrate, acetate, and propionate is an important mechanism through which the gut microbiome regulates the immune system.^76,37^ Once the bacterial SCFA is absorbed in T lymphocytes, SCFAs regulate the fate of T cells through activation of G protein-coupled receptors (GPR) such as GPR41/GPR43 signaling cascade, inhibition of histone deacetylases, and alteration of metabolic status by upregulating the activity of the mammalian target of rapamycin (mTOR) complex.^37^ Such interaction between T cell immunometabolism and SCFAs lead to mucosal T_regs_ expansion, inflammatory cascades inhibition, reduced production of anti-inflammatory cytokine such as interleukin (IL)-10 and an increased production of pro-inflammatory cytokine such as interferon-γ (IFN-γ).^37,54^ Gut microbiome dysbiosis caused by prolonged deviation from the microbial homeostasis may lead to intestinal inflammation and consequently development of autoimmunity.^55^ This can induce pro-inflammatory environment in the intestinal lumen as well as the mucosa by increasing the production of the inflammatory cytokines (TNFα, IFNγ, IL1β, IL-6, and IL-17), and translocation of microbial products to the gastrointestinal tract and adjacent organs. Subsequently, the disrupted immunity maturation and appearance of self-antigens lead to immune-mediated diseases such as T1D.^55^

## IMPACT OF T1D ON GUT MICROBIAL DIVERSITY

Diversity is a measure utilized to describe the complexity of a microbial community. It is defined as the variety and abundance of species in a defined unit of study. Microbiome diversity can be measured by **α-**diversity and β-diversity scales. **α-**diversity describe as evenness and richness within a habitant unit. Species richness measures the number of functionally related taxa observed in the community regardless of their frequencies whereas evenness represents the similarity and equitability of proportions of taxa frequencies in a community.^56^ β-diversity is defining the expression of the similarity between the microbial community.^56^

The associations between gut microbiome diversity and T1D are well documented in the literature though few studies do not find such an association.^27,39,46,47,57–61^ A study conducted in Finland showed that the level of microbial diversity diminishes overtime and the development of T1D in young children with T1D-associated autoimmunity.^58^ In another study, Finnish children were recruited from two intervention trials and the composition of intestinal microbiota in autoantibody-negative children was compared with children who have at least two diabetes-associated autoantibodies.^47^ In this study, the bacterial diversity diminishes in autoantibody-positive children when compared with autoantibody-negative children overtime.^57^ The results of this study was in agreement with a previous study which reported decreased microbial diversity with the development of T1D and increasing age.^58^ Another study examined 33 HLA- matched infants from birth until 3 years old to assess the alterations in the composition of gut microbiome as T1D develops. The community diversity decreased significantly in children who progress to T1D after seroconversion (a change from seronegative to a seropositive condition) but before disease diagnosis.^59^ Further, a marked 25% drop in **α**-diversity observed in T1D progressors compared to controls. The decline in **α-**diversity accompanied by a spike in inflammation-associated species, alterations to metabolic pathways and proinflammatory environment after seroconversion but before T1D onset.^59,62^ Emerging evidence indicate that **α-**diversity, β-diversity and community richness are dissimilar in T1D vs healthy control subjects.^27,39,47,61^ Children with diabetes demonstrated a notably lower gut microbiota diversity and richness compared to control groups.^27^ This is consistent to another study which reported different patterns of β-diversity clustering in children with T1D compared to healthy controls.^47^ Further, higher loss of diversity of the dominant bacterial community in T1D children might be pertinent to the autoimmune process.^47^ Studies conducted in Northern Italy reported a lower **α-**diversity in adolescents with T1D and 27% less diversity in children with T1D and as compared to healthy controls.^60,61^ The above studies reported a signficant decrease in the microbial diversity in children genetically predisposed to T1D and children with T1D. However, few studies did not find such an association between T1D and microbial diversity.^15,31,45,46,63,64^ German BABYDIET study cohort which analyzed stool samples from children within the first 3 years of their life did not find significant differences in bacterial diversity between islet cell autoantibody–positive and anti-islet cell autoantibody–negative children.^63^ In a Southeast Sweden study, the effect of HLA alleles on the human gut microbiome composition and diversity was assessed. The authors suggested that HLA does not affect gut microbiome diversity considering the lack of significant differences in diversity between risk groups.^15^ Findings from a study of four geographically distant African and Asian countries demonstrated that α-diversity and β-diversity measures did not show appreciable differences between children with T1D and healthy controls, which is aligned with the results of a study conducted in Mexican children.^46,64^ In a recent prospective cohort study conducted in three different centers in Australia observed no differences in α and β-diversity between children with T1D, children with 2 or more auto antibodies, sibling and unrelated controls.^31^

## IMPACT OF T1D ON GUT MICROBIAL TAXONOMIC PROFILES

Gut microbiota is composed of microorganisms such as bacteria, archeae, fungi, yeast, and viruses. Bacterial classification includes the major taxonomic ranks such as phyla, classes, orders, families, genera, and species. Recent studies analyzed the microbial profile at various taxonomic and community levels to identify specific taxa associated with T1D in children. Evidence from these studies indicate that there is a significant difference in the microbial community at different taxonomic level exist in between healthy children and children with T1D.^45,58,65^

### Alterations at the phyla level

Actinobacteria, Bacteroidetes, Firmicutes, Fusobacteria, Proteobacteria, and Verrucomicrobia are considered the dominant gut microbial phyla. The two phyla Firmicutes and Bacteroidetes represent 90% of gut microbiota.^66^ The gut microbiome composition of children is dominated by Bacteroidetes and Firmicutes, followed by Actinobacteria and Proteobacteria.^27,39,45,47,61,67^

#### Actinobacteria

Actinobacteria, one of the largest bacterial phyla are Gram-positive bacteria with high guanine + cytosine (G + C) DNA content and mainly represented by the *Bifidobacterium* genus.^66^ *Bifidobacterium* genus presented in the human gut have a significant role in maintaining health not only within the gastrointestinal tract but in the rest of the body.^68^ *Bifidobacterium* genera contribute to butyrate production and inhibiting bacterial translocation.^68^ Human studies show that the gut microbiome composition is different in children with T1D and healthy controls. At the phylum level, the bacterial number of Actinobacteria was shown to decrease significantly in children with T1D compared to healthy children.^45,47^ However, another study conducted in Finland reported higher abundance of Actinobacteria in children with T1D.^44^

#### Bacteroidetes

Bacteroidetes phylum shown to exhibit diabetogenic properties and belong to gram-negative bacteria.^39^ Bacteroidetes impair the barrier function of the epithelial cells which favors chronic inflammation.^49^ Specifically *Bacteroides* genus which belong to Bacteroidetes play a key role in the development of T1D possibly through glutamate decarboxylase production which might provokes glutamic acid decarboxylase autoimmunity via molecular mimicry.^39^ Bacteroides acquires a substantial amount of antibiotics resistance genes and hence an increased antibiotic usage especially in developed countries leads to Bacteroides over abundance.^61,69^ Studies showed a successive increase in Bacteroidetes abundance at phylum level in children with T1D and children who develop T1D over time.^45,58,65^ In contrast, few studies indicate that the Bacteroidetes were found to be less abundant in healthy control children as they become more abundant in T1D children.^39,57,69^ Two studies conducted on Finish children analyzed the gut microbial composition in children who had HLA-conferred susceptibility to T1D and tested positive for at least two diabetes-associated autoantibodies and matched healthy control children.^46,61^ In this study, abundance of Bacteroidetes phylum was different in autoantibody-positive children before, at, and after islet autoantibody seroconversion. In addition, the Bacteroidetes were more common in autoantibody-positive children with respect to autoantibody negative matches.^57,58^

#### Firmicutes

Firmicutes belong to gram-negative bacteria and one of the major phyla that has the most common organisms in human gut microbiota. The intestinal microbiota of healthy people is dominated by Firmicutes phyla.^27,45^ It is well documented that the proportions of Firmicutes phyla are higher in healthy control group as compared to children with T1D.^44,45,47,58,69^ Indeed, the Firmicutes sequences convey an inverse pattern in the microbiome composition of the children with T1D. Firmicutes sequences decline overtime in T1D whereas increase in healthy children.^58^ A recent study analyzed the gut microbiome in four different groups that include newly diagnosed T1D children, children who tested positive for one to four autoantibodies, seronegative first-degree relatives and healthy controls.^65^ The abundance of Firmicutes were different among the four groups suggesting the possible link between seropositive group with more than one autoantibody and alteration in the abundance of Firmicutes bacterium.^65^ These results are consistent with two other studies conducted in Finland.^57,58^

#### Firmicutes/Bacteroidetes ratio

Firmicutes and Bacteroidetes are the most abundant taxa of gut microbiome despite their huge inter-individual variations and dramatic dynamics.^70^ Studies indicate that the distribution ratio of the most abundant phyla such as Bacteroidetes, Firmicutes and Actinobacteria are different between healthy children and children with T1D.^45,58,65^ Indeed, children develop autoimmune diabetes overtime as the Firmicutes abundance decline and Bacteroidetes abundance increase.^27,58,61,64,65^ Human and animal studies suggested Firmicutes to Bacteroidetes ratio (F/B ratio) and Bacteroidetes to Firmicutes ratio (B/F ratio) as an index of the health of gut microbes.^70,71^ Evidence suggest that there is a correlation between seropositivity with more than one autoantibody and alteration in the proportion of bacteria within the Bacteroidetes and Firmicutes phyla.^65^ Further, F/B ratio was significantly different between children who develop T1D and healthy children. Studies indicate that B/F ratio significantly increase over time in children with T1D and children who eventually progressed to clinical T1D whereas B/F ratio decrease in nondiabetic children.^45,47,58^ However, two studies performed in Mexican and Chinese children did not find any significant difference in B/F ratio between children with T1D and healthy children.^27,46^ Hence, the influence of F/B or B/F ratio on T1D is not well established and yet to be studied.

#### Proteobacteria

Proteobacteria is one of the major phyla belongs to gram negative bacteria. Studies that assessed the association between the abundance of Proteobacteria and T1D reported contradictory results. Two studies showed a significant positive association between Proteobacteria and T1D while other studies reported a significantly higher abundance of Proteobacteria in control groups compared to T1D.^44,47,64,69^ One study did not find any association between the relative abundance of Proteobacteria between healthy individuals and T1D patients.^45^

### Alterations at the genus level

Emerging evidence indicates major alterations in the bacterial composition at the genus level between healthy individuals and children and adolescent with T1D.^44,58,61^ These studies showed that the children with T1D presented with higher abundance of 12 genera including *Escherichia, Bacteroides, Clostridium, Veillonella, Ruminococcus, Blautia, Streptococcus, Sutterella, Enterobacter, Lactobacillus, Lactococcus*, and *Bifidobacterium*.^27,39,44–47,58,60,64,69^ Despite the immense variability of the gut microbiota in children with T1D regardless of the confounding variables (geography, age, ethnicity, and diet), by far *Bacteroides* is the dominant genus reported in most published studies.^46,47^ *Bacteroides* is a gram-negative, acetate- and propionate-producing bacterium, which contribute to chronic inflammation.^49^ It is speculated that *Bacteroides* genera provokes dysbiosis, epithelial cell barrier dysfunction and consequently T1D.^44,46,49^ A study which investigated the role of *Bacteroides* by comparing the microbiome composition of autoantibody-positive and autoantibody-negative revealed complementary results.^57^ The investigators showed an increase in the abundance of *Bacteroides* in autoantibody-positive male children compared to healthy children.^57^ Further, one study which sought to determine the composition of the gut microbiome in children who developed anti-islet cell autoimmunity, they did not find the difference in the abundance of *Bacteroides* between autoantibody-positive and autoantibody-negative children after adjusting for confounding factors and correction for multiple testing. However, they hypothesized that children with autoantibody-positive presented with compromised bacterial network which could potentially be associated with the development of anti-islet cell autoimmunity.^63^ The gastrointestinal of the healthy children is enriched with genera such as *Prevotela, Akkermansia, Bifidobacterium, Lachnospira*, and the butyrate producer genera including *Roseburia, Anaerostipes, Faecalibacterium, Eubacterium*, and *Subdoligranulum*.^44–47,58,64^ Specifically, the proportion of *Prevotella* and *Akkermansia* was shown to have 20 and 140 fold higher abundance in healthy children compared to children with T1D, respectively.^44^ The overabundance of butyrate producer and mucin degraders in the gut microbiome of healthy children is the indicator of the beneficial effects of these genera on gut integrity.^44,72^

### Alterations at the species level

Children with T1D exhibit altered gut microbiome composition at the species level as compared to their healthy peers. Apparently, the gut microbiome evolution of diabetic children is one step behind with aberrant direction.^39^ Shotgun metagenomics studies provide evidence for the alterations of gut microbiome at species level. A spike in *Bacteroides clarus, Alistipes obesi, Bifidobacterium longum, Dialister invisus, Gemella sanguinis, Bifidobacterium longum, Clostridium stercorarium, Ruminococcus gnavus* and *Streptococcus infantarius* was reported in T1D children.^39,52,59,61^ A study aimed to investigate the gut microbiome development in children with higher genetic risk for T1D in Finland showed that the *Bacteroides dorei* and *Bacteroides vulgatus* are the dominant species in T1D.^69^ It was suggested that *Bacteroides dorei* produce a lipopolysaccharide which has immunoinhibitory properties that contribute to the development of T1D by preventing early immune development.^73^ A different study showed contradictory results which were thought to be attributed to variation due to age, geographical regions and different metabolic function of the same bacteria determined by its methylation patterns.^67,74^ A study that investigated the differences in the intestinal microbiota composition of children with at least two diabetes-associated autoantibodies and autoantibody-negative children revealed correlations with β-cell autoimmunity at the species level.^57^ This study reported that the abundance of the important short-chain fatty acid–producing species including *Bifidobacterium adolescentis, B. Pseudocatenulatum, Faecalibacterium prausnitzii, Clostridium clostridioforme*, and *Roseburia faecis* are inversely associated with the number of diabetes-associated autoantibodies in children implicating four autoantibodies acquire significantly less short-chain fatty acid producer species.^57^ *Clostridium clusters IV* and *XIVa* are butyrate-producing species found to be higher in healthy children as compared to diabetic children.^39,64^ Consistently, a recent study in Finland found that *Clostridium clusters IV* and *XIVa* were negatively correlated with the number of diabetes-specific autoantibodies in Finish children.^57^ A study in which the stool samples were collected at three different time points revealed striking results.^58^ In this study, the gut microbiome of the children who eventually developed T1D overtime enriched with bacterium *mpn-isolate group 18, Bacteroides ovatus, Bacteroides sp. CJ78, Bacteroides thetaiotaomicron* and *Bacteroides uniformis* species.^58^ Further, *Bacteroides ovatus* species composes approximately 24% of the total increase in T1D children as compared to healthy controls. These findings are in line with another study which confirms the overabundance of *Bacteroides ovatus* in the diabetic children.^69^ On the contrary, in healthy control group *Bacteroides fragilis, Bacteroides. vulgatus, Eubacterium eligens, Eubacteriumrectale, Faecalibacterium prausnitzii*, human intestinal *firmicute CB47* and human intestinal *firmicute CO19* species were observed to be significantly higher in comparison with the T1D diabetic individuals.^58^ Of those species, the human *firmicute strain CO19* comprised nearly 20% of the increase as to cases overtime.^58^ A study conducted in Turkey showed an increase in *Enterobacteriaceae spp* colonization with an decrease in *Bifidobacterium spp*. in T1D children as compared to healthy individuals.^75^ Previous study confirms these findings emphasizing the importance of butyrate-producing bacteria in the β-cell autoimmunity development.^57^ The Environmental Determinants of Diabetes (TEDDY) study was conducted in six clinical research centers including in United States (Colorado, Georgia/Florida, Washington) and in Europe (Finland, Germany and Sweden) .^23^ The primary goal of this prospective cohort study was to determine the environmental causes of T1D. They collected monthly samples from 783 children at the age of three months until they develop islet autoimmunity or T1D.^23^ In this study, T1D children had higher abundance of *Bifidobacterium pseudocatenulatum, Roseburiahominis* and *Alistipes shahii* species whereas the healthy individuals presented higher abundance of *Streptococcus thermophilus* and *Lactococcus lactis* species. They also found geographical variations in the overabundance of different species for instance, higher level of *Streptococcus mitis/oralis/pneumoniae* species in Finish children with T1D, higher level of *Streptococcus thermophilus* in Colorado healthy individuals, and higher level of *Bacteroides vulgatus* in Swedish T1D children.^23^ A recent study in Italian children and adolescents features over abundance of *Bifidobacterium stercoris, Bacteroides intestinalis, Bacteroides cellulosilyticus*, and *Bacteroides fragilis* species in T1D patients and *Bacteroides vulgatus* in helathy controls.^60^ All these evidence justifies the importance and the need for considering the geographical location while studying the gut microbiome composition.

## IMPACT OF T1D ON THE FUNCTIONAL POTENTIAL OF GUT MICROBIOME

A limited number of studies conducted the metagenomic analysis to map the differences in the functional potential of gut microbiome between individuals with T1D and healthy individuals.^23,44,47,48,59,60,73^ These studies reported the impact of T1D on the alteration in metabolic pathways. Healthy individuals have more functionally diverse microbiome composition compared to T1D and healthy controls have eight times more functions with greater abundance.^44^ The bacterial fermentation pathways involve in the production of SCFA such as butyrate, acetate and propionate.^77^ A case-control comparison in TEDDY study reported an increase in bacterial fermentation pathways in healthy controls compared to individuals with T1D.^23^ Specifically, the degradation of L-arginine, putrescine, 4-aminobutanoate, acetylene, l-1,2-propanediol and the fermentation of acetyl coenzyme A pathways that are involved in the biosynthesis of butyrate, acetate, propionate and butanoate were more abundant in healthy controls.^23^ The findings of the TEDDY study confirm existing evidence regarding the protective role of SCFAs in T1D in human.^39,57^ A study conducted in Finland assessed metabolic pathways in samples collected from healthy controls and from children after presenting with two autoimmune antibodies.^44^ In this study, a robost functional difference in 911 functions was observed in between the two groups out of the total 3,849 functions identified. Further, 797 functions were prevalent in healthy children and 114 functions were more prevalent in children presenting autoimmune antibodies. Interestingly, the samples collected from children presenting autoimmune antibodies exhibited different functionality between each other and healthy controls. However, all healthy controls exhibited similar functions. Further, the authors identified 1,061 pathways using Kyoto Encyclopedia of Genes and Genomes (KEGG) maps which is a reference database for pathway mapping. In this analysis, 166 pathways were significantly more prevalent in healthy controls while only 24 pathways were statistically prevalent in children presenting autoimmune antibodies. This study also support the previous studies which showed a great level of butyrate production in healthy individuals.^45,78^ In healthy controls the major functional categories including carbohydrate metabolism, amino acid metabolism, DNA Metabolism, RNA metabolism, cell wall and capsule proteins, nucleotides and nucleosides, cofactors and vitamins, motility and chemotaxis, nitrogen metabolism, membrane transport, phosphorous metabolism, virulence, and respiration expressed significantly higher relative abundance of read.^44^ The samples collected from children presenting autoantibodies showed lower functional diversity indicating that their microbiome composition possesses fastidious bacteria which require more nutrient in the external environment to survive and grow.^44^

Phylogenetic Investigation of Communities by Reconstruction of Unobserved States (PICRUSt) is a bioinformatics software which predicts metagenome function from marker gene (e.g. 16s rRNA) surveys. PICRUSt analysis in previous studies indicated that numerous bacterial functions were over- or underrepresented in individuals with diabetes vs healthy individuals related to their different microbiome composition.^47,60^ An impairment in the pathways related to glucose metabolism and iron complex levels was predicted in Italian children and adolecents with T1D.^60^ Another study predicted that the abundance of genes related to energy and carbohydrate metabolism pathways deplete in T1D group compared to healthy controls.^47^ However, the genes linked with lipid metabolism and amino acid metabolism, LPS biosynthesis, arachidonic acid metabolism, ATP-binding-cassette transport, antigen processing and presentation, and chemokine signaling pathways related to inflammation and immune response were overrepresented in T1D.^47^ Further, this study demonstrated a significant increase in LPS, proinflammatory cytokines (IL-1β, IL-6, TNF-**α)** and a significant depletion of anti-inflammatory cytokines (IL-10 and IL-13) in subjects with T1D. This situation together with lower abundance of anti-inflammatory bacteria, promotes dysregulation of epithelial integrity and autoimmune responses in T1D.^47,79^

## KNOWLEDGE GAPS, CHALLENGES AND FUTURE DIRECTION

Gut dysbiosis is implicated in a number of diseases and understanding the gut microbiome composition should be one of the priorities for the prevention and treatment of metabolic diseases such as diabetes. Despite recent developments in the field of the gut microbiome, there are significant knowledge gaps in understanding the role of gut microbiome in T1D especially in children and adolescents. In addition, the alterations in the diversity and relative abundance of gut microbes reported in T1D are not consistent in some of the studies (Table 1). This could be because of number of factors that could influence the microbiome data which includes but not limited to participants (geographical location, age, gender, dietary pattern, physical activity, severity of T1D, etc.), methods used for sample collection, DNA isolation method, microbial profiling method (16 s rRNA gene amplification and shotgun metagenomics), and the bioinformatics tools used for the analysis. Hence, these factors should be considered in the future studies.

Future studies should focus on novel approaches to understand the association between gut microbes and T1D, and to develop microbiome-based therapeutic strategies for the prevention and treatment of diabetes. (1) Based on the current knowledge, it is challenging to identify whether the alteration of gut microbiota is a cause and/or consequence of T1D. As discussed previously, a study assessed HLA-matched infants from birth until 3 years old to determine the alterations in the composition of gut microbiome as T1D develops. This type of studies should be expanded to identify whether alteration of gut microbiota is a cause or consequence of T1D. (2) Evidence indicate microbial metabolites such as SCFAs modulate the development or prevention of T1D. In addition to SCFAs, gut microbes mediate the production of number of metabolites. Future studies should focus on identifying the impact of these microbial metabolites on T1D and the molecular mechanisms involved. (3) The current knowledge about the functional potential of gut microbiome and the gut microbiota at species level are limited in T1D. It is important to identify the gut microbiome composition at species level and determine the functional potential of gut microbiome by using latest high throughput sequencing technologies such as Shotgun metagenomics. (4) Identifying specific microbe(s) involved in the pathogenesis of T1D will also lead to the early detection of T1D in genetically susceptible children. (5) Emerging evidence indicates the role of gut virome in T1D but the studies are limited. Studies focusing on T1D and gut microbes should include virome analysis. (6) The composition and functional potential of gut microbiome is extensively altered by a number of factors including diet, age, gender, geographical location, genetic background, hygiene, antibiotic use, and other medical practices. These factors should be considered when analyzing the data as they may skew the results of microbiome data. Further, future studies should consider controlling the possible experimental variables. (7) Geographical location has a major impact on the alteration in gut microbiome. Understanding the composition of the gut microbiome in a specific geographical location is necessary to develop microbiome-based therapeutic strategies tailored based on location. (8) Identifying the effect of diet and physical activity on gut microbiome is also important for developing microbiome-based therapeutic strategies. (9) Finally, future intervention studies should focus on diets that contain the probiotics and/or prebiotics to prevent the development of T1D. This can lead to the development of effective microbiome-based strategy to complement existing traditional therapies for treating and/or preventing diabetes in children and adolescents.
